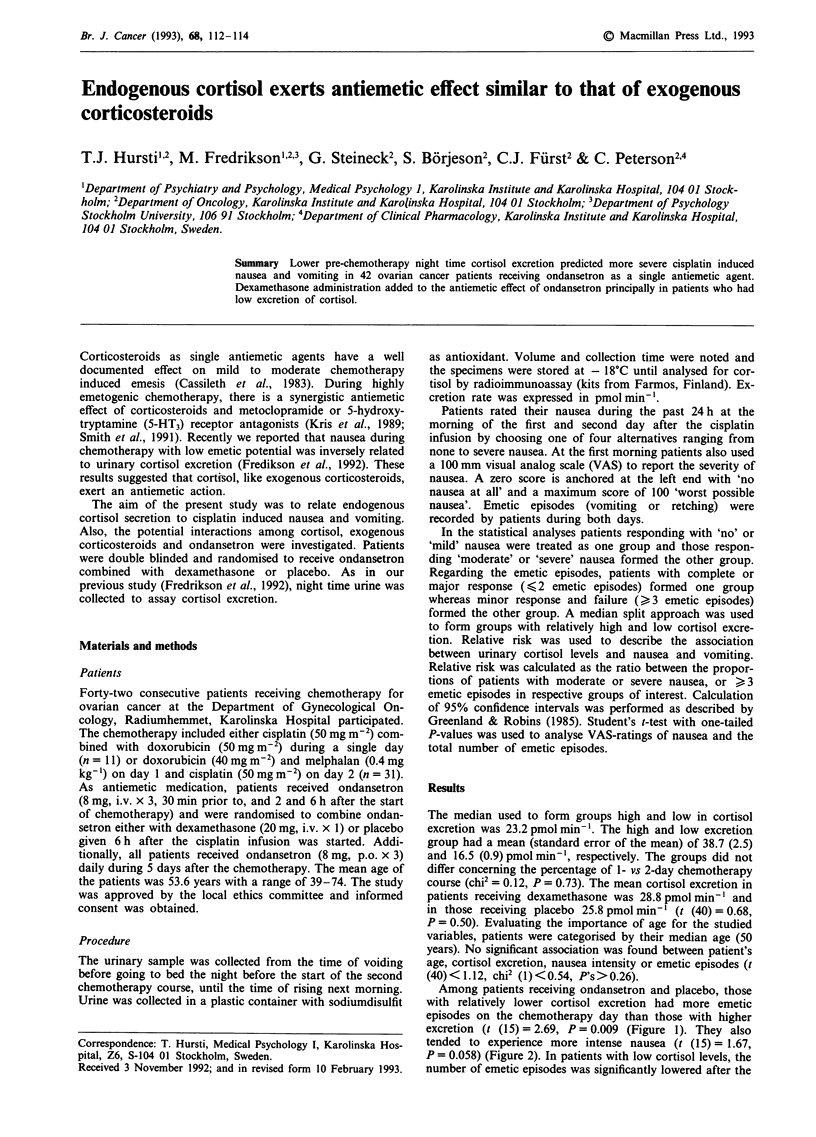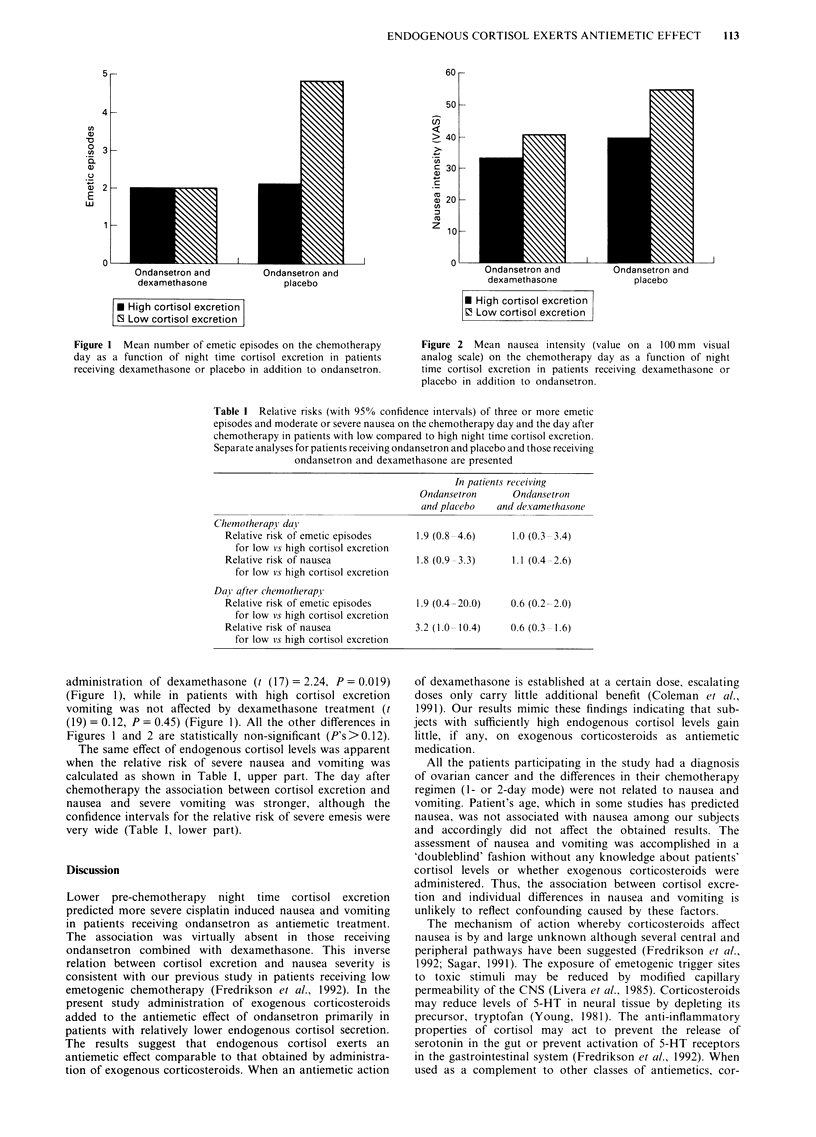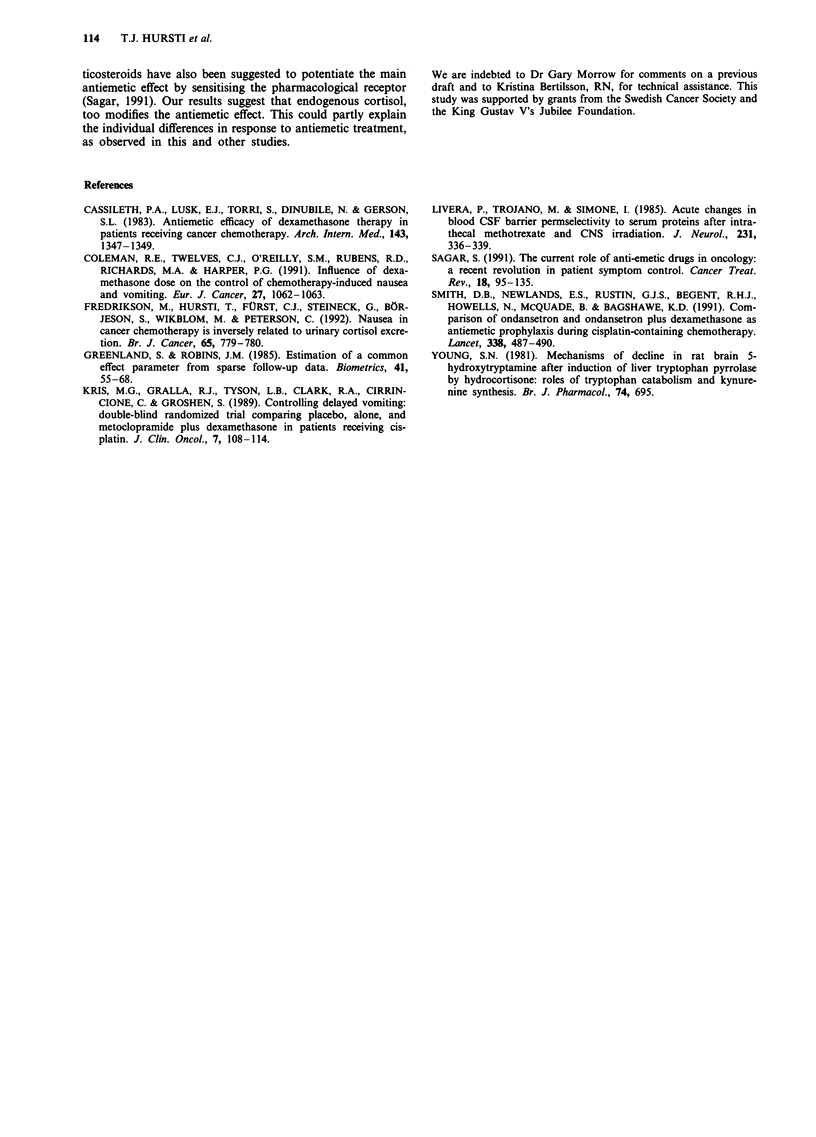# Endogenous cortisol exerts antiemetic effect similar to that of exogenous corticosteroids.

**DOI:** 10.1038/bjc.1993.295

**Published:** 1993-07

**Authors:** T. J. Hursti, M. Fredrikson, G. Steineck, S. Börjeson, C. J. Fürst, C. Peterson

**Affiliations:** Department of Psychiatry and Psychology, Karolinska Institute, Stockholm, Sweden.

## Abstract

Lower pre-chemotherapy night time cortisol excretion predicted more severe cisplatin induced nausea and vomiting in 42 ovarian cancer patients receiving ondansetron as a single antiemetic agent. Dexamethasone administration added to the antiemetic effect of ondansetron principally in patients who had low excretion of cortisol.


Br. J. Cancer (1993), 68, 112–114 © Macmillan Press Ltd., 1993

### Endogenous cortisol exerts antiemetic effect similar to that of exogenous corticosteroids

T.J. Hursti[1,2], M. Fredrikson[1,2,3], G. Steineck[2], S. Börjeson[2], C.J. Fürst[2] & C. Peterson[2,4]

[1]*Department of Psychiatry and Psychology, Medical Psychology 1, Karolinska Institute and Karolinska Hospital, 104 01 Stockholm;* [2]*Department of Oncology, Karolinska Institute and Karolinska Hospital, 104 01 Stockholm;* [3]*Department of Psychology Stockholm University, 106 91 Stockholm;* [4]*Department of Clinical Pharmacology, Karolinska Institute and Karolinska Hospital, 104 01 Stockholm, Sweden.*

**Summary** Lower pre-chemotherapy night time cortisol excretion predicted more severe cisplatin induced nausea and vomiting in 42 ovarian cancer patients receiving ondansetron as a single antiemetic agent. Dexamethasone administration added to the antiemetic effect of ondansetron principally in patients who had low excretion of cortisol.

Corticosteroids as single antiemetic agents have a well documented effect on mild to moderate chemotherapy induced emesis (Cassileth *et al.*, 1983). During highly emetogenic chemotherapy, there is a synergistic antiemetic effect of corticosteroids and metoclopramide or 5-hydroxytryptamine ($5\text{-}HT_3$) receptor antagonists (Kris *et al.*, 1989; Smith *et al.*, 1991). Recently we reported that nausea during chemotherapy with low emetic potential was inversely related to urinary cortisol excretion (Fredikson *et al.*, 1992). These results suggested that cortisol, like exogenous corticosteroids, exert an antiemetic action.

The aim of the present study was to relate endogenous cortisol secretion to cisplatin induced nausea and vomiting. Also, the potential interactions among cortisol, exogenous corticosteroids and ondansetron were investigated. Patients were double blinded and randomised to receive ondansetron combined with dexamethasone or placebo. As in our previous study (Fredrikson *et al.*, 1992), night time urine was collected to assay cortisol excretion.

### Materials and methods

### Patients

Forty-two consecutive patients receiving chemotherapy for ovarian cancer at the Department of Gynecological Oncology, Radiumhemmet, Karolinska Hospital participated. The chemotherapy included either cisplatin ($50\ mg\ m^{-2}$) combined with doxorubicin ($50\ mg\ m^{-2}$) during a single day ($n = 11$) or doxorubicin ($40\ mg\ m^{-2}$) and melphalan ($0.4\ mg\ kg^{-1}$) on day 1 and cisplatin ($50\ mg\ m^{-2}$) on day 2 ($n = 31$). As antiemetic medication, patients received ondansetron (8 mg, i.v. × 3, 30 min prior to, and 2 and 6 h after the start of chemotherapy) and were randomised to combine ondansetron either with dexamethasone (20 mg, i.v. × 1) or placebo given 6 h after the cisplatin infusion was started. Additionally, all patients received ondansetron (8 mg, p.o. × 3) daily during 5 days after the chemotherapy. The mean age of the patients was 53.6 years with a range of 39–74. The study was approved by the local ethics committee and informed consent was obtained.

### Procedure

The urinary sample was collected from the time of voiding before going to bed the night before the start of the second chemotherapy course, until the time of rising next morning. Urine was collected in a plastic container with sodiumdisulfit as antioxidant. Volume and collection time were noted and the specimens were stored at − 18°C until analysed for cortisol by radioimmunoassay (kits from Farmos, Finland). Excretion rate was expressed in $pmol\ min^{-1}$.

Patients rated their nausea during the past 24 h at the morning of the first and second day after the cisplatin infusion by choosing one of four alternatives ranging from none to severe nausea. At the first morning patients also used a 100 mm visual analog scale (VAS) to report the severity of nausea. A zero score is anchored at the left end with 'no nausea at all' and a maximum score of 100 'worst possible nausea'. Emetic episodes (vomiting or retching) were recorded by patients during both days.

In the statistical analyses patients responding with 'no' or 'mild' nausea were treated as one group and those responding 'moderate' or 'severe' nausea formed the other group. Regarding the emetic episodes, patients with complete or major response ($\leqslant 2$ emetic episodes) formed one group whereas minor response and failure ($\geqslant 3$ emetic episodes) formed the other group. A median split approach was used to form groups with relatively high and low cortisol excretion. Relative risk was used to describe the association between urinary cortisol levels and nausea and vomiting. Relative risk was calculated as the ratio between the proportions of patients with moderate or severe nausea, or $\geqslant 3$ emetic episodes in respective groups of interest. Calculation of 95% confidence intervals was performed as described by Greenland & Robins (1985). Student's *t*-test with one-tailed *P*-values was used to analyse VAS-ratings of nausea and the total number of emetic episodes.

### Results

The median used to form groups high and low in cortisol excretion was $23.2\ pmol\ min^{-1}$. The high and low excretion group had a mean (standard error of the mean) of 38.7 (2.5) and 16.5 (0.9) $pmol\ min^{-1}$, respectively. The groups did not differ concerning the percentage of 1- *vs* 2-day chemotherapy course ($chi^2 = 0.12$, $P = 0.73$). The mean cortisol excretion in patients receiving dexamethasone was $28.8\ pmol\ min^{-1}$ and in those receiving placebo $25.8\ pmol\ min^{-1}$ ($t\ (40) = 0.68$, $P = 0.50$). Evaluating the importance of age for the studied variables, patients were categorised by their median age (50 years). No significant association was found between patient's age, cortisol excretion, nausea intensity or emetic episodes ($t\ (40) < 1.12$, $chi^2\ (1) < 0.54$, $P\text{'s} > 0.26$).

Among patients receiving ondansetron and placebo, those with relatively lower cortisol excretion had more emetic episodes on the chemotherapy day than those with higher excretion ($t\ (15) = 2.69$, $P = 0.009$ (Figure 1). They also tended to experience more intense nausea ($t\ (15) = 1.67$, $P = 0.058$) (Figure 2). In patients with low cortisol levels, the number of emetic episodes was significantly lowered after the

Correspondence: T. Hursti, Medical Psychology I, Karolinska Hospital, Z6, S-104 01 Stockholm, Sweden.
Received 3 November 1992; and in revised form 10 February 1993.

**Figure 1** Mean number of emetic episodes on the chemotherapy day as a function of night time cortisol excretion in patients receiving dexamethasone or placebo in addition to ondansetron.

**Figure 2** Mean nausea intensity (value on a 100 mm visual analog scale) on the chemotherapy day as a function of night time cortisol excretion in patients receiving dexamethasone or placebo in addition to ondansetron.

**Table I** Relative risks (with 95% confidence intervals) of three or more emetic episodes and moderate or severe nausea on the chemotherapy day and the day after chemotherapy in patients with low compared to high night time cortisol excretion. Separate analyses for patients receiving ondansetron and placebo and those receiving ondansetron and dexamethasone are presented

| | *In patients receiving* | |
|---|---|---|
| | *Ondansetron and placebo* | *Ondansetron and dexamethasone* |
| *Chemotherapy day* | | |
| Relative risk of emetic episodes for low *vs* high cortisol excretion | 1.9 (0.8–4.6) | 1.0 (0.3–3.4) |
| Relative risk of nausea for low *vs* high cortisol excretion | 1.8 (0.9–3.3) | 1.1 (0.4–2.6) |
| *Day after chemotherapy* | | |
| Relative risk of emetic episodes for low *vs* high cortisol excretion | 1.9 (0.4–20.0) | 0.6 (0.2–2.0) |
| Relative risk of nausea for low *vs* high cortisol excretion | 3.2 (1.0–10.4) | 0.6 (0.3–1.6) |

administration of dexamethasone ($t$ (17) = 2.24, $P = 0.019$) (Figure 1), while in patients with high cortisol excretion vomiting was not affected by dexamethasone treatment ($t$ (19) = 0.12, $P = 0.45$) (Figure 1). All the other differences in Figures 1 and 2 are statistically non-significant ($P$'s > 0.12).

The same effect of endogenous cortisol levels was apparent when the relative risk of severe nausea and vomiting was calculated as shown in Table I, upper part. The day after chemotherapy the association between cortisol excretion and nausea and severe vomiting was stronger, although the confidence intervals for the relative risk of severe emesis were very wide (Table I, lower part).

### Discussion

Lower pre-chemotherapy night time cortisol excretion predicted more severe cisplatin induced nausea and vomiting in patients receiving ondansetron as antiemetic treatment. The association was virtually absent in those receiving ondansetron combined with dexamethasone. This inverse relation between cortisol excretion and nausea severity is consistent with our previous study in patients receiving low emetogenic chemotherapy (Fredrikson *et al.*, 1992). In the present study administration of exogenous corticosteroids added to the antiemetic effect of ondansetron primarily in patients with relatively lower endogenous cortisol secretion. The results suggest that endogenous cortisol exerts an antiemetic effect comparable to that obtained by administration of exogenous corticosteroids. When an antiemetic action of dexamethasone is established at a certain dose, escalating doses only carry little additional benefit (Coleman *et al.*, 1991). Our results mimic these findings indicating that subjects with sufficiently high endogenous cortisol levels gain little, if any, on exogenous corticosteroids as antiemetic medication.

All the patients participating in the study had a diagnosis of ovarian cancer and the differences in their chemotherapy regimen (1- or 2-day mode) were not related to nausea and vomiting. Patient's age, which in some studies has predicted nausea, was not associated with nausea among our subjects and accordingly did not affect the obtained results. The assessment of nausea and vomiting was accomplished in a 'doubleblind' fashion without any knowledge about patients' cortisol levels or whether exogenous corticosteroids were administered. Thus, the association between cortisol excretion and individual differences in nausea and vomiting is unlikely to reflect confounding caused by these factors.

The mechanism of action whereby corticosteroids affect nausea is by and large unknown although several central and peripheral pathways have been suggested (Fredrikson *et al.*, 1992; Sagar, 1991). The exposure of emetogenic trigger sites to toxic stimuli may be reduced by modified capillary permeability of the CNS (Livera *et al.*, 1985). Corticosteroids may reduce levels of 5-HT in neural tissue by depleting its precursor, tryptofan (Young, 1981). The anti-inflammatory properties of cortisol may act to prevent the release of serotonin in the gut or prevent activation of 5-HT receptors in the gastrointestinal system (Fredrikson *et al.*, 1992). When used as a complement to other classes of antiemetics, corticosteroids have also been suggested to potentiate the main antiemetic effect by sensitising the pharmacological receptor (Sagar, 1991). Our results suggest that endogenous cortisol, too modifies the antiemetic effect. This could partly explain the individual differences in response to antiemetic treatment, as observed in this and other studies.

We are indebted to Dr Gary Morrow for comments on a previous draft and to Kristina Bertilsson, RN, for technical assistance. This study was supported by grants from the Swedish Cancer Society and the King Gustav V's Jubilee Foundation.

**References**

CASSILETH, P.A., LUSK, E.J., TORRI, S., DINUBILE, N. & GERSON, S.L. (1983). Antiemetic efficacy of dexamethasone therapy in patients receiving cancer chemotherapy. *Arch. Intern. Med.*, **143**, 1347–1349.

COLEMAN, R.E., TWELVES, C.J., O'REILLY, S.M., RUBENS, R.D., RICHARDS, M.A. & HARPER, P.G. (1991). Influence of dexamethasone dose on the control of chemotherapy-induced nausea and vomiting. *Eur. J. Cancer*, **27**, 1062–1063.

FREDRIKSON, M., HURSTI, T., FÜRST, C.J., STEINECK, G., BÖRJESON, S., WIKBLOM, M. & PETERSON, C. (1992). Nausea in cancer chemotherapy is inversely related to urinary cortisol excretion. *Br. J. Cancer*, **65**, 779–780.

GREENLAND, S. & ROBINS, J.M. (1985). Estimation of a common effect parameter from sparse follow-up data. *Biometrics*, **41**, 55–68.

KRIS, M.G., GRALLA, R.J., TYSON, L.B., CLARK, R.A., CIRRINCIONE, C. & GROSHEN, S. (1989). Controlling delayed vomiting: double-blind randomized trial comparing placebo, alone, and metoclopramide plus dexamethasone in patients receiving cisplatin. *J. Clin. Oncol.*, **7**, 108–114.

LIVERA, P., TROJANO, M. & SIMONE, I. (1985). Acute changes in blood CSF barrier permselectivity to serum proteins after intrathecal methotrexate and CNS irradiation. *J. Neurol.*, **231**, 336–339.

SAGAR, S. (1991). The current role of anti-emetic drugs in oncology: a recent revolution in patient symptom control. *Cancer Treat. Rev.*, **18**, 95–135.

SMITH, D.B., NEWLANDS, E.S., RUSTIN, G.J.S., BEGENT, R.H.J., HOWELLS, N., MCQUADE, B. & BAGSHAWE, K.D. (1991). Comparison of ondansetron and ondansetron plus dexamethasone as antiemetic prophylaxis during cisplatin-containing chemotherapy. *Lancet*, **338**, 487–490.

YOUNG, S.N. (1981). Mechanisms of decline in rat brain 5-hydroxytryptamine after induction of liver tryptophan pyrrolase by hydrocortisone: roles of tryptophan catabolism and kynurenine synthesis. *Br. J. Pharmacol.*, **74**, 695.